# Integrative genomic and transcriptomic analysis of genetic markers in Dupuytren’s disease

**DOI:** 10.1186/s12920-019-0518-3

**Published:** 2019-07-11

**Authors:** Junghyun Jung, Go Woon Kim, Byungjo Lee, Jong Wha J. Joo, Wonhee Jang

**Affiliations:** 1 Department of Life science, Dongguk University-Seoul, Seoul, 04620 Republic of Korea; 2 Department of Pharmacology, College of Pharmacy, Kyung Hee University, 26 Kyungheedae-ro, Seoul, 02447 South Korea; 3 Department of Computer Science and Engineering, Dongguk University-Seoul, Seoul, 04620 South Korea

**Keywords:** Dupuytren’s disease, Unfolded protein response (UPR), Endoplasmic reticulum (ER) stress, Multiple-phenotype analysis, *trans*-regulatory hotspots, ZFP57 zinc finger protein, Major histocompatibility complex class II

## Abstract

**Background:**

Dupuytren’s disease (DD) is a fibroproliferative disorder characterized by thickening and contracting palmar fascia. The exact pathogenesis of DD remains unknown.

**Results:**

In this study, we identified co-expressed gene set (DD signature) consisting of 753 genes via weighted gene co-expression network analysis. To confirm the robustness of DD signature, module enrichment analysis and meta-analysis were performed. Moreover, this signature effectively classified DD disease samples. The DD signature were significantly enriched in unfolded protein response (UPR) related to endoplasmic reticulum (ER) stress. Next, we conducted multiple-phenotype regression analysis to identify *trans*-regulatory hotspots regulating expression levels of DD signature using Genotype-Tissue Expression data. Finally, 10 *trans*-regulatory hotspots and 16 eGenes genes that are significantly associated with at least one *cis*-eQTL were identified.

**Conclusions:**

Among these eGenes, major histocompatibility complex class II genes and ZFP57 zinc finger protein were closely related to ER stress and UPR, suggesting that these genetic markers might be potential therapeutic targets for DD.

**Electronic supplementary material:**

The online version of this article (10.1186/s12920-019-0518-3) contains supplementary material, which is available to authorized users.

## Background

Dupuytren’s disease (DD) is a fibroproliferative disorder characterized by palmar fascia hypertrophy that often results in thickening and contracting palmar fascia [1]. DD mostly occurs in ring finger, followed by little and middle fingers, where affected fingers become permanently and irreversibly bent in a flexed position [2]. The prevalence of DD rises with increasing age and DD is most commonly seen in Europe. It has higher prevalence in northern Europe than that in southern Europe [3]. Even though Lee et al. have recently shown that DD is not a disease limited to European descent anymore, it is still classified as a rare and hard-to-care disease in Korea [4]. Alcoholism, smoking, dyslipidemia, and diabetes are regarded as risk factors of DD; however, the exact etiopathogenesis of DD remains unclear [5].

With rapid growth of high-throughput technology, previous studies have reported that several genes are associated with the progression of DD based on differentially expressed gene (DEG) analysis using microarray [6–11]. Most DEG studies were focused on single genes without considering interconnections between genes, especially those with high number of connections (edges) in a network. Co-expression network analysis based on similar expression patterns can be effectively used for identifying a set of genes that are simultaneously active in the same functional processes [12]. Together with transcriptomic data, several risk genetic loci related to the pathogenesis of DD have been identified by genome-wide association studies (GWAS) to inspect the association of a single phenotype and each single nucleotide polymorphism (SNP) [13–15]. However, SNPs cannot solely explain biological processes because most of such variants reside in noncoding regions of the genome [16].

Recently, large quantities of genomic data alongside with expression data per individual have been gathered from GWAS cohorts. The Genotype-Tissue Expression (GTEx) project was founded for expression quantitative trait locus (eQTL) mapping, which investigates effects of genetic variation on gene expression in extensively diverse primary tissues from human [17]. eQTL analysis indeed is an approach to explain genetic variation underlying altered gene expression [18]. Recent studies have described tissue-specific eQTLs because gene expression patterns are different across tissues [19, 20]. *cis*-eQTLs or *trans*-eQTLs usually refers to eQTLs that regulate nearby or distal genes, respectively [18], and genes that are significantly associated with at least one *cis*-eQTL are referred to as eGenes [21]. Notably, previous eQTL studies showed that a small number of genomic regions referred to as *trans*-regulatory hotspots can regulate expression levels of hundreds of genes [22–24], suggesting the existence of master regulators of transcription. Typically, eQTL approaches analyzing independent phenotypes have low statistical power [25]. On the other hand, multi-variate methods analyzing many phenotypes simultaneously can increase the power to identify underlying regulatory hotspots in a complex biological system [26].

In this study, we used weighted gene co-expression network analysis (WGCNA) to find co-expression gene set (module) of highly correlated genes for DD. Using independent datasets as validation sets, we confirmed the reliability of selected gene set via module enrichment analysis based on gene set enrichment analysis (GSEA) and disease classification. Finally, a multiple-phenotype regression analysis was performed using GTEx muscle data to identify regulatory hotspots related to gene set of DD.

## Methods

### Microarray preprocessing and meta-analysis

Affymetrix microarray datasets were preprocessed and normalized following Jung et al. [27]. Other platform datasets including CodeLink, Stanford, and Illumina platform were preprocessed using limma R package [28]. Two-color microarray dataset (GSE2688) comparing relative expression levels between a sample RNA and a universal RNA in a single microarray was adjusted for batch effects after independent normalization because the dataset was from two types of array platforms. Meta-analysis was conducted using the one-color microarray datasets measuring expression levels from each sample separately (Table 1). These datasets were combined using unique Entrez IDs. Meta-analysis was carried out according to SVA R package [29] after adjusting for batch effects using Combat [30].

**Table 1:** Characteristics of NCBI GEO datasets used for WGCNA analysis

	GEO series ID	Array type	Array platform	No. of arrays (DD: Control)	Source of tissue	PMID
1	GSE2688 (GSE4457)	Two-color	Stanford Microarray	11 (4: 7)	DD tissues	18694919 [6], 16473681 [7]
2	GSE21221	One-color	GE Healthcare CodeLink Human Whole Genome Bioarray	12 (6: 6)	Fibroblasts derived from DD tissues	18433489 [40]
3	GSE31356	One-color	Affymetrix Human Genome U133A Array	6 (3: 3)	DD tissues	22965824 [8]
4	GSE41524	One-color	Affymetrix Human Exon 1.0 ST Array	10 (4: 6)	Fibroblasts derived from DD tissues	23554969 [9]
5	GSE59746	One-color	Affymetrix Human Genome U133 Plus 2.0 Array	4 (2: 2)	DD tissues	25379672 [10]
6	GSE75152	One-color	Illumina HumanWG-6 v3.0 expression beadchip	24 (12: 12)	DD tissues	27467239 [11]

### Weighted gene correlation network analysis

GSE75152 dataset contained mRNA expression profiles of 12 DD patients and 12 control subjects with total RNA extracted from the connective tissue from the hand [11]. Top 5000-most expressed probes were selected for computational cost and simplicity after normalization. Multiple probes representing one gene were collapsed using collapseRows function [31]. A signed WGCNA was used to identify co-expression modules comprised of positively correlated genes based on Pearson correlation coefficient [32]. In detail, a similarity matrix based on Pearson correlation of all pairs of genes was converted into an adjacency matrix via a power function. A suitable soft-thresholding power (β) of the power function was selected via analysis of scale-free topology. Next, the adjacency matrix was transformed into a topological overlap matrix (TOM) to reflect topological information of a network. Modules were defined by a hybrid tree cut method when cutting a hierarchical cluster trees [32]. Expression patterns in modules were summarized by module eigengene. The threshold of minimum size of modules was 50 genes and pairs of modules with high ME correlations (r > 0.85) were merged.

### Module enrichment analysis and functional annotation

The fast preranked gene set enrichment analysis (fgsea) R package was used for GSEA [33]. Microarrays of log fold change (log FC) values were regarded as a pre-ranked list. Modules derived from WGCNA were used as gene sets for module enrichment analysis. Database for Annotation, Visualization and Integrated Discovery (DAVID) [34] was used to conduct functional enrichment analysis.

### Disease classification

Random forest [35] classification was conducted using classification and regression training (caret) r package [36]. Combined one-color microarray dataset and two-color microarray dataset were transformed to mean 0 and variance 1 for each gene. Model training for DD sample classification was performed with the one-color microarray dataset using LOOCV. Model validation was carried out with the two-color microarray dataset.

### Regulatory hotspot analysis

To identify *trans*-regulatory hotspots, we performed GAMMA, one of multiple-phenotype analysis approaches to examine an association between a number of phenotypes or gene expression levels and each SNP [25]. GAMMA is preferred over other multiple-phenotype approaches as it is scalable to high dimensional data, containing hundreds to thousands number of genes which is often the case with eQTL data. In addition, utilizing linear mixed model, it considers widely known genetic relatedness referred to as population structure in the data. The population structure complicates association analysis by inducing spurious signals. Especially, in multiple-phenotype analysis, these problems may compound as bias because population structure accumulates from each phenotype [25]. Skeletal muscle data from 361 samples in GTEx project (version 6) data in dbGaP database (accession phs000424.v6.p1) were used for this analysis. The top three principal components of covariates were regressed out in expression data. GAMMA was performed by an adaptive permutation which increased the number of permutations from 10^2^ to 10^6^, increasing by 10 folds each time. A summary data of SNP-gene associations was obtained from the GTEx Portal (http://gtexportal.org). SNPs within ±1 Mb of the transcriptional start site of each gene were used to identify *cis*-eQTL.

### Cross-species mapping using ER stress gene expression data

In this study, gene expression microarray dataset GSE35209 obtained from MEFs treated with an ER stress-inducing agent (tunicamycin) was used. The pre-processed data were obtained using GEOquery r package [37]. Cross-species mapping between human and mouse genes was performed using National Center for Biotechnology Information HomoloGene database (Build68) [38].

## Results

### Identification of co-expression module for DD

**Fig. 1:**
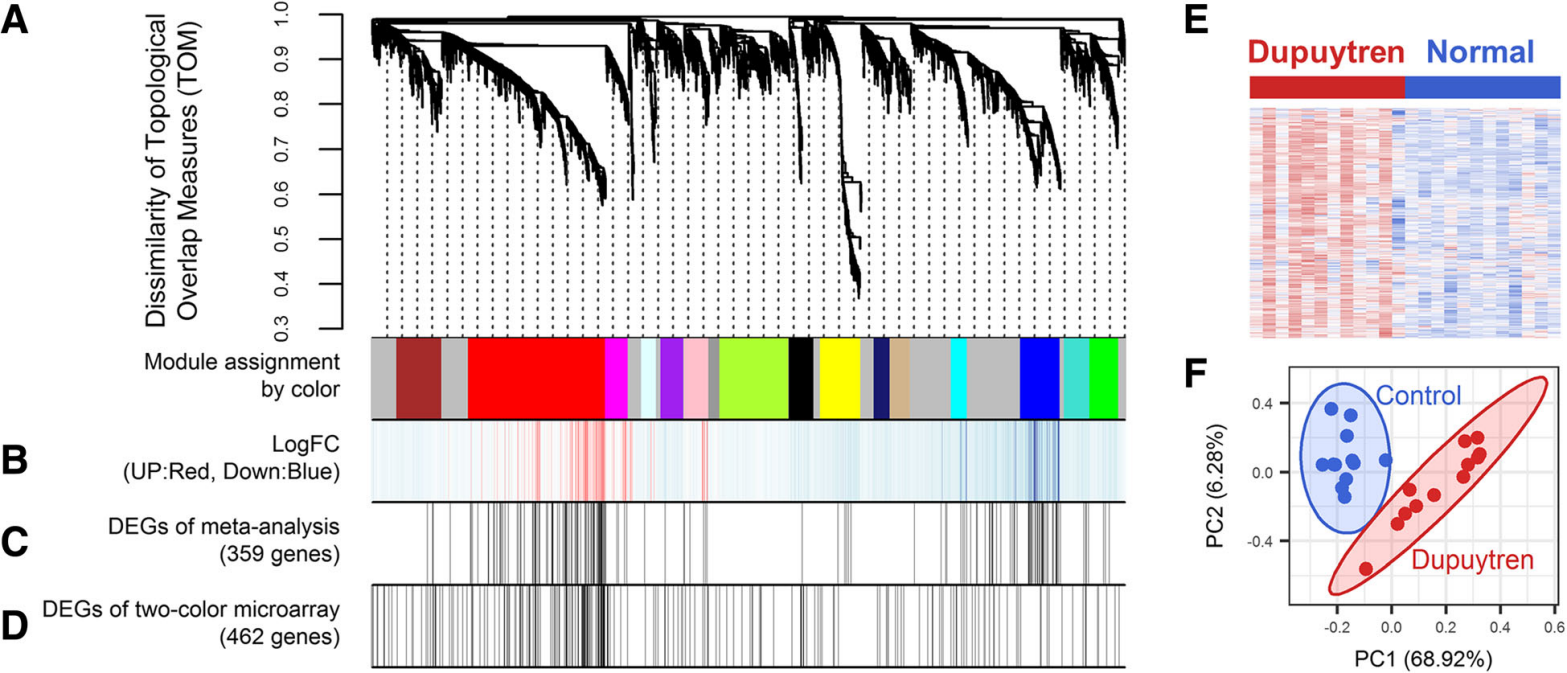
Identification of modules associated with gene expression of DD. **a** Dendrogram showing modules based on the dissimilarity of TOM (1-TOM). Color bars below show assignment of modules. **b** Heatmap showing the gene expression pattern of modules. Red and blue color lines indicate up- and down-regulation, respectively. **c** A Heatmap showing the results of the meta-analysis derived from one-color microarray dataset. Black indicates DEGs (FDR < 0.01). **d** A Heatmap showing results of DEGs analysis of two-color microarray dataset. Black color lines indicate DEGs (FDR < 0.01). **e** A Heatmap showing expression patterns of genes for red module. Black color lines indicate DEGs (FDR < 0.01). **f** A scatter plot of principal component analysis (PCA) showing a distinct separation of the expression level of red module genes between patients with DD and normal subjects

We constructed co-expression networks using the WGCNA r package describing correlation patterns among genes across DD patients and normal subjects to identify a representative set of genes for DD. Among the top 5000-most expressed microarray probes in GSE75152, we identified unique 4141 representative genes based on collapseRows function in WGCNA r package [31]. Selected genes with similar expression patterns were clustered into gene set modules via average linkage hierarchical cluster analysis. The power of β = 12 (scale-free R^2^ = 0.82) was selected as the soft-thresholding power for co-expression network construction (Additional file 1). We identified 16 co-expression modules representing genes that shared highly similar expression patterns (Fig. 1a). Among these modules, the red and blue modules were enriched in up- and down-regulated genes, respectively (Fig. 1b). Next, we carried out meta-analysis using one-color microarray datasets to identify representative module for DD. A total of 67 microarray data in five independent studies were used, consisting of 31 DD samples and 36 normal samples (Table 1). The results showed that DEGs derived from meta-analysis were significantly enriched in red (Two-sided Fisher’s exact test: odds ratio = 2.98 and *P* value = 2.64e-23) and blue (Two-sided Fisher’s exact test: odds ratio = 3.96 and *P* value = 1.23e-14) modules (Fig. 1c and Additional file 2A). Additionally, DEGs of two-color microarray datasets were also significantly enriched in red (Two-sided Fisher’s exact test: odds ratio = 2.50 and *P* value = 6.35e-19) and blue modules (Two-sided Fisher’s exact test: 0.35, odds ratio = 0.35 and *P* value = 6.49e-4) (Fig. 1d and Additional file 2B).

### DD signature has the power to classify DD samples

**Fig. 2:**
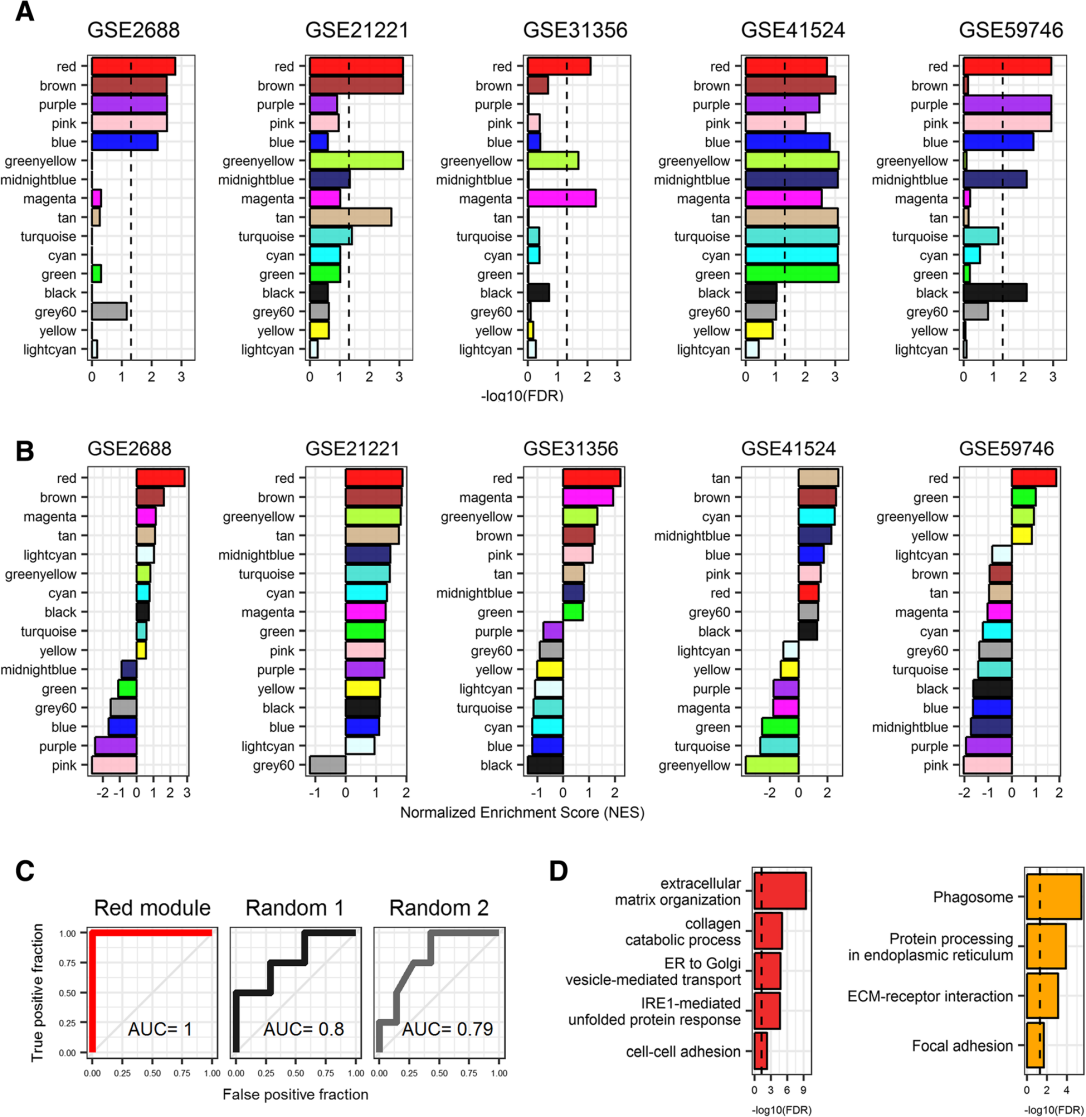
Robustness of DD signature and its functional annotations. **a** Bar plots showing the results of module enrichment analysis using GSEA. Black dotted lines indicate significant threshold (FDR < 0.05). **b** Bar plots showing normalized enrichment score (NES) for results of module concentration analysis using GSEA. **c** ROC analysis with AUC showing the performance of classification using red module in (**a**). Randomly selected genes consist of the same number of the red module gene. **d** Bar plots showing the results of functional enrichment analysis. Red and orange color bars represent GO biological process and KEGG pathway, respectively. Black dotted lines indicate significant threshold (FDR < 0.05)

To test whether these identified modules were replicated in other 5 independent datasets (Table 1), GSEA were performed for module enrichment analysis. The results showed that only the red module (753 genes) was significantly and positively enriched in all 5 independent datasets (FDR < 0.05) (Fig. 2a and b). Consistently, the expression levels of red module genes were distinct and discriminative according to the DD patient or the normal subject data in GSE75152 (Fig. 1e and f). We then asked whether red module genes could sufficiently classify disease state of individual samples related to DD. In order to apply a sample classification approach of DD samples, all datasets including CodeLink, Affymetrix, and Illumina were merged (Table 1). Among the 753 red module genes, only 255 genes remained after merging the datasets because genes contained by each microarray platforms are different. Model training using random forest method was carried out with one-color microarray datasets using Leave-one-out cross-validation (LOOCV) while model validation was performed using two-color microarray dataset. The Classification performance showed that area under the curve (AUC) value in receiver operating characteristic (ROC) analysis curve was higher for red module genes than that of two randomly selected genes having the same number of red module gene (Fig. 2c). Collectively, these results strongly suggested that the red module genes (so-called DD signature in this paper) (Additional file 3) were a robust set of genes representing DD.

### Functional enrichment analysis of DD signature revealed dysregulated functions in DD

To obtain insights into the biological process of DD, we performed functional enrichment analysis to define dysregulated Gene Ontology (GO) categories and Kyoto Encyclopedia of Genes and Genomes (KEGG) pathway of the DD signature. We found terms for extracellular matrix (ECM) or collagen were significantly enriched in extracellular matrix organization (GO:0030198 and FDR = 5.97e-10), ECM-receptor interaction (hsa04512 and FDR = 7.60e-04), and collagen catabolic process (GO:0030574 and FDR = 4.62e-06), while the enriched terms for adhesion were cell-cell adhesion (GO:0098609 and FDR = 1.41e-03) and focal adhesion (hsa04510 and FDR = 2.01e-02) (Fig. 2d and Additional file 4). Previous results from microarray studies indicated that several proteins for extracellular matrix (ECM) are correlated with mRNA dysregulation [6, 39–41] and that pathological collagen deposition is related to DD [42]. Notably, unfolded protein response (UPR) related to endoplasmic reticulum (ER) stress terms were significantly enriched in protein processing in endoplasmic reticulum (hsa04141 and FDR = 1.34e-04) and IRE1-mediated unfolded protein response (GO:0036498 and FDR = 2.06e-05) (Fig. 2d and Additional file 4). UPR and ER stress have been detected in many disease including neurodegenerative disease, cancer, diabetes, liver disorders, and obesity [43]. Our results suggest that unfolded protein-induced ER stress can also be involved in the pathogenesis of DD.

### Identification of regulatory hotspot related to DD signature

**Fig. 3:**
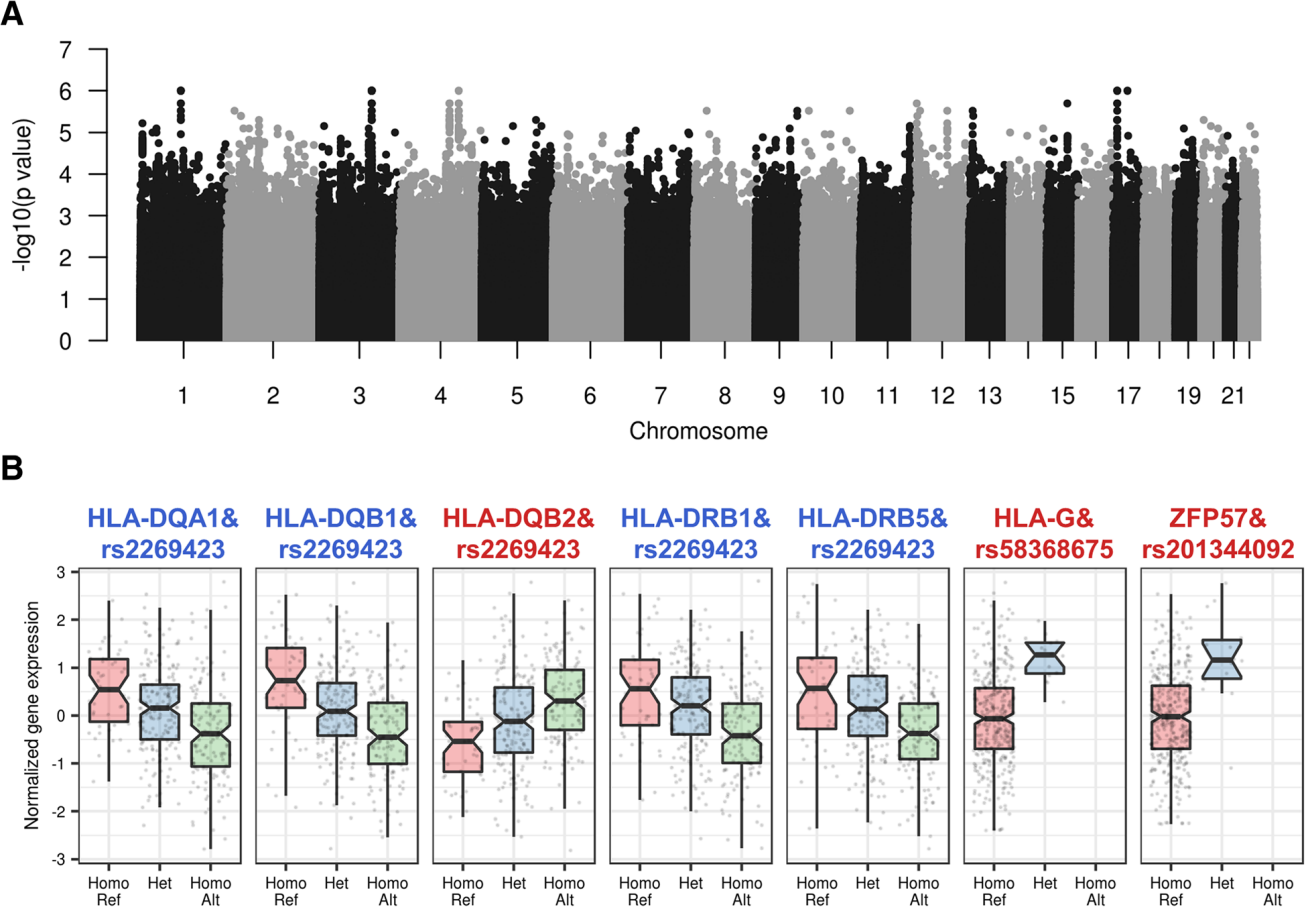
Identification of *trans*-regulatory hotspots associated with DD signature. **a** The GAMMA results applied to GTEx dataset using DD signature. **b** Box plots of gene expression levels of eGenes by each *trans*-regulatory hotspot between the different genotype groups

**Fig. 4:**
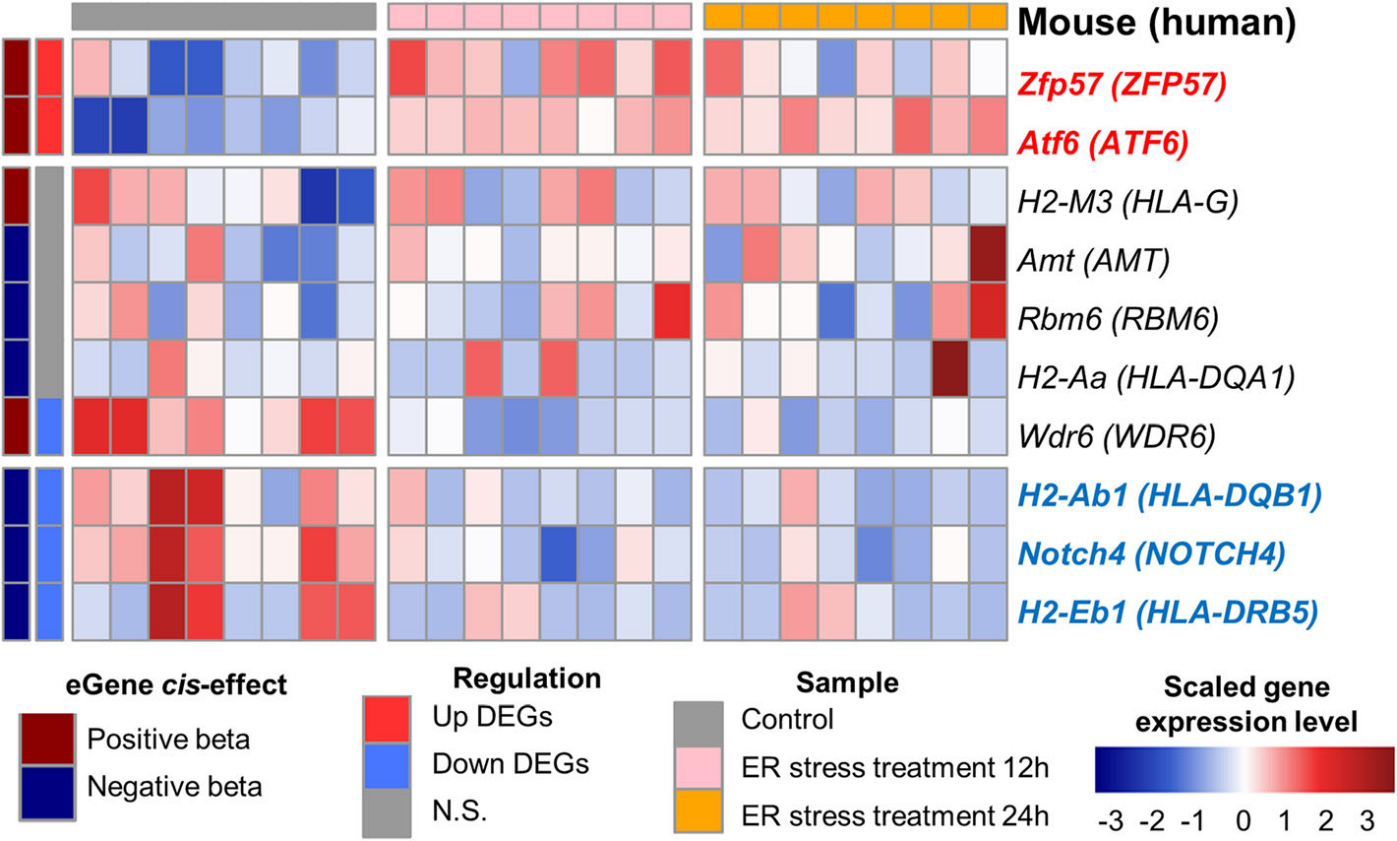
A Heatmap showing gene expression levels of eGene using microarray data derived from ER stress-inducing agent (tunicamycin) treated MEFs. Color bars give information on tunicamycin treatment condition, DEGs, and direction of effect of SNP. Mouse gene symbol (human gene symbol) was represented by row names of the heatmap

To identify *trans*-regulatory hotspots regulating expression levels of DD signature, multiple-phenotype regression analysis was carried out using GAMMA (Generalized analysis of molecular variance for mixed-model analysis) [25] in eQTL datasets of GTEx version 6 dataset. The presence of abnormal myofibroblasts in DD palmar fascia plays a causative role in digital contracture of DD [44]. Therefore, muscle tissues were used in this analysis. First, we found 512 loci using GAMMA (*P* value <5e-05) (Fig. 3a). We then examined *cis*-acting SNPs within ±1 Mb region of the transcription start site (TSS) of each gene among GAMMA loci because recent studies indicated that expression change of a *trans*-acting factor by a *cis*-eQTL was another possible causal mechanism [45, 46]. Finally, we identified 10 GAMMA loci and 16 eGenes (q value < 0.05) that were significantly associated with at least one *cis*-acting SNP [21] (Table 2), including 5 protein-encoding eGenes: major histocompatibility complex class (MHC) II DQ alpha 1 (*HLA-DQA1*), DQ beta 1 (*HLA-DQB1*), DQ beta 2 (*HLA-DQB2*), DR beta 1 (*HLA-DRB1*), and DR beta 5 (*HLA-DRB5*). Their expression levels were significantly associated with one rs2269423 on chromosome 6 (Fig. 3b and Table 2). Previous results showed that human leukocyte alleles (HLA) encoding MHC proteins in humans, especially HLA-DR alleles, are related to DD [47, 48]. Among the identified 5 HLA genes related to rs2269423, 4 genes were also identified to have significant variant-gene associations in GTEx version 7 dataset, and 3 genes including HLA-DR beta 6 (*HLA-DRB6*), *NOTCH4*, and activating transcription factor 6 beta (*ATF6B*) were additionally identified (Additional file 5 and Fig. 4). ATF6 is a key transcription factor for unfolded protein response (UPR) pathway during ER stress [49]. rs201344092 allele was associated with an increase in gene expression of *ZFP57* (Fig. 3b, c and Table 2). Importantly, a mutation of *ZFP57* is associated with neonatal diabetes type 1 [50]. Previous studies indicated that *Zfp57* expression was up-regulated by ER stress condition in mice [51, 52], suggesting that *Zfp57* might be secondary UPR-regulated transcriptional repressor [53].

**Table 2:** List of significant associations with DD signature. The lowest GAMMA *P* for SNP was listed among multiple SNP related to one eGene (See also Additional file 4)

Chr	Position	rs ID	GAMMA *P* value		eGenes (q value < 0.05)		
				Gene Symbol	Gene Name	Effect size (beta)	Gene Type
3	49,769,419	rs62262722	1.4E-05	AMT	aminomethyltransferase	−0.16	protein coding
				FAM212A	family with sequence similarity 212 member A	0.26	protein coding
	49,858,661	rs55997059	1.7E-05	WDR6	WD repeat domain 6	0.20	protein coding
	49,898,318	rs62260755	2.4E-05	RBM6	RNA binding motif protein 6	−0.28	protein coding
6	30,500,730	rs148983519	3.8E-05	HCG9	HLA complex group 9 (non-protein coding)	1.11	lincRNA
	30,642,417	rs201344092	1.1E-05	ZFP57	ZFP57 zinc finger protein	1.44	protein coding
	30,664,568	rs58368675	2.2E-05	HLA-G	major histocompatibility complex, class I, G	1.34	protein coding
	32,145,707	rs2269423	1.2E-05	HLA-DQA1	major histocompatibility complex, class II, DQ alpha 1	−0.44	protein coding
				HLA-DRB5	major histocompatibility complex, class II, DR beta 5	−0.41	protein coding
				HLA-DQB2	major histocompatibility complex, class II, DQ beta 2	0.46	protein coding
				HLA-DQB1	major histocompatibility complex, class II, DQ beta 1	−0.51	protein coding
				HLA-DRB1	major histocompatibility complex, class II, DR beta 1	−0.42	protein coding
				HLA-DQB1-AS1	HLA-DQB1 antisense RNA 1	−0.54	antisense
12	9,799,363	rs58481733	4.4E-05	DDX12P	DEAD/H-box helicase 12, pseudogene	−0.57	pseudogene
15	40,374,582	rs73390668	2.9E-05	SRP14-AS1	SRP14 antisense RNA1 (head to head)	−0.31	lincRNA
	77,312,826	rs16968627	4.4E-05	RP11-797A18.4	RP11-797A18.4	−0.55	lincRNA

### Expression of eGenes under ER stress condition

To determine whether expression levels of the identified genes are regulated by ER stress condition, we examined the gene expression levels using microarray data derived from mouse embryonic fibroblasts (MEFs) treated with ER stress-inducing agent (GSE35209). 10 genes including 2 up-regulated and 4 down-regulated DEGs remained after cross-species mapping procedure. Remarkably, *Zfp57* (*ZFP57* in human) was significantly up-regulated in ER stress condition (Fig. 4) and *ZFP57* is positively regulated by the *trans*-regulatory hotspot (rs201344092) associated with DD signature in human (Fig. 3b). *H2-Eb1* (*HLA-DRB5* in human) and *H2-Ab1* (*HLA-DQB1* in human) were significantly down-regulated in ER stress condition (Fig. 4) and *HLA-DRB5* and *HLA-DQB1* are negatively regulated by the *trans*-regulatory hotspot (rs2269423) associated with DD signature in human (Fig. 3b). Together, our data suggest that the regulatory hotspot nearby *ZFP57* and MHC class II genes may develop ER stress condition by regulating ER stress-s and UPR-related genes.

## Discussion

The number of patients with DD has continued to increase from 1118 in 2007 to 3280 in 2014 in the United States [54]. A surgical treatment including fasciotomy with or without dermofasciectomy is the current treatment option for DD [47]. However, surgical treatment has a high recurrence rate [54]. It has been reported that collagenase clostridium histolyticum (CCH) injection approved by Food and Drug Administration in 2010 is more effective and safer treatment option than surgical strategy, although the long-term recurrence rate of this CCH injection approach has not been fully determined yet [47]. Understanding the pathogenesis of DD is important to find out novel nonsurgical approach for DD treatment.

Although a recent study showed that DD is not a disease limited to European descent anymore [4], DD is still not commonly found in African Americans and Asians than in Europeans [55]. A total of 6 datasets were found in the public database (Table 1), due to the rareness of the disease. We carried out WGCNA to find co-expression modules based on Pearson correlation for gene expression similarities of DD. To construct the co-expression network, we used GSE75152 which is the largest dataset used in this study (Table 1). Because WGCNA was performed based on Pearson correlation, more samples could lead to more robust the results [56]. We used signed WGCNA that created biologically meaningful modules than unsigned WGCNA [12]. After identifying co-expressed modules, module enrichment analysis was carried out. The red module genes were enriched in all 5 datasets (Fig. 2a and b) although the datasets were heterogeneous, consisting of two types of tissue sources and different microarray platforms with small sample size. The results suggested that red module genes were representative and robust genes for DD. Moreover, the WGCNA may tend to classify DEGs into certain modules due to the higher between-group variance which can translate to stronger correlations. That is why only red module was significant in the five independent datasets. The pre-filtering based on top 5000-most expressed microarray probes and limitation of 50 gene size of the modules were used for WGNCA. The prefiltering for reducing noise and the minimizing module size for module functional annotation both seemed to be appropriate because the red module genes were enriched in the biological process of unfolded protein-induced ER stress, which was not emphasized in previous studies.

GAMMA is a multiple-phenotype analysis method that examines an association between each SNP and multiple phenotypes or gene expression levels, while simultaneously correcting for population stratification utilizing linear mixed model [25]. We identified a total of 10 regulatory hotspots in muscle eQTL dataset of GTEx project using gene expression levels of DD signature. The identified regulatory hotspots included *HLA-DQA1*, *HLA-DQB1*, *HLA-DQB2*, *HLA-DRB1*, *HLA-DRB5*, and *ZFP57*. HLA is known to be associated with various human diseases, including rheumatoid arthritis, multiple sclerosis, Crohn’s disease, type 1 diabetes (T1D), and HIV [57]. Intriguingly, HLA regions are known as the strongest genetic determinants in T1D, contributing up to 50% of the genetic risk to T1D susceptibility [58]. Diabetes is one of the known risk factors in DD and has been reported that approximately 20% of diabetic patients have DD [59]. DD is also involved in a constellation of musculoskeletal diseases affecting hand associated with diabetes [60]. T1D and DD seem to be inherited together rather than diabetes being an etiological risk factor for DD. Along with a previous report on association of HLA-DRB1 and HLA-DQB1 with T1D patients [61], it can be considered that having altered SNPs in HLA region might lead to genetic susceptibility in both DD and T1D, thus explaining the association of these two conditions.

Recently, *ZFP57* has been identified as a candidate gene contributing to HLA associated diseases including cancers, autoimmune diseases, and HIV [62]. *ZFP57* is located in HLA region of chromosome 6 and acts as a transcriptional factor that *trans*-regulates genomic imprinting, especially during development [63, 64]. Notably, transient neonatal diabetes (TND), an early onset T1D, are primarily caused by aberrant expression of imprinted genes due to mutations in *ZFP57* [65].

To date, alterations in extracellular matrix proteoglycan organization and collagen overproduction are the two main mechanisms proposed for the development of DD [9, 66]. Apart from these known pathological factors, we newly found the involvement of ER stress in DD. Accumulation of unfolded or misfolded proteins in ER under various pathophysiological conditions is defined as ER stress. ER stress and ER stress-responsive genes have been implicated in numerous diseases including neurological diseases, cancers, and diabetes [43]. It has been reported that expression of HLA molecules is decreased under ER stress conditions such as palmitate or glucose starvation and tunicamycin treatment [67]. Previous microarray results from MEFs identified that expression of *ZFP57* is altered after treatment with ER stress inducer [51]. Based on previous studies and our results, it can be concluded that ER stress-induced transcriptional changes in *ZFP57* and HLA molecules are implicated in the disease phenotype of DD. Thus, an in-depth investigation on the connection of ER stress with DD, possibly in relation to *ZFP57* and HLA, is required in order to understand pathophysiology of DD.

Because DD was classified as a rare disease amongst Eastern Asians, there were substantial difficulties in studying DD until recently. In such cases, an integrative analysis using genomic and transcriptomic data can serve as a powerful tool to study the pathogenesis, individual susceptibility, and progression of the disease. This study has some limitations. First, a further experimental step is needed to validate the identified targets. Second, we only focused on skeletal muscle data when identifying regulatory hotspots although subcutaneous fat and fibroblast are also known to be related to DD [47]. Despite these limitations, we successfully identified robust genetic markers of DD, suggesting that they may be potential therapeutic targets.

## Conclusions

DD is a fibroproliferative disorder in thickening and contracting palmar fascia with unknown etiopathogenesis. In this study, we identified DD signature and potential cause of regulatory hotspots for DD based on integrative genomic and transcriptomic analysis using multiple phenotype regression analysis and WGCNA. Module enrichment analysis and classification analysis was used to determine the robustness of the identified markers. Finally, we identified MHC class II genes and ZFP57 were closely related to ER stress and UPR, suggesting that these genetic markers might be potential therapeutic targets for DD.

## Additional files

Additional file 1: Identification of soft-thresholding power for co-expression network construction. (A) An analysis of scale free topology for picking an appropriate soft-thresholding power. (B) An analysis of the mean connectivity for picking an appropriate soft-thresholding power. (PPTX 277 kb)

Additional file 2: Point range plots showing the results of the enrichment of DEGs about each module via Fisher’s exact test. (A) Odds ratios for the enrichment of DEGs compared with non-DEGs by one-color meta-analysis with 95% confidence intervals of odds. Because tan and midnight blue modules have no DEGs, the odds ratio is zero. (B) Odds ratios for the enrichment of DEGs compared with non-DEGs by two-color microarray analysis and 95% confidence intervals of the odds. (PPTX 242 kb)

Additional file 3: The identified red module genes (DD signature). (XLS 120 kb)

Additional file 4: The results of functional enrichment analysis in Fig. 2d. (XLS 38 kb)

Additional file 5: Violin plots showing the results of significant variant-gene associations related to rs2269423 in GTEx version 7. (PPTX 155 kb)

## Abbreviations

AUC: Area under the curve
DD: Dupuytren’s disease
ECM: Extracellular matrix
eQTL: Expression quantitative trait locus
ER: Endoplasmic reticulum
GO: Gene Ontology
GWAS: Genome-wide association studies
HLA: Human leukocyte alleles
KEGG: Kyoto Encyclopedia of Genes and Genomes
LOOCV: Leave-one-out cross-validation
MEF: Mouse embryonic fibroblast
MHC: Major histocompatibility complex
ROC: Receiver operating characteristic
SNP: Single nucleotide polymorphism
T1D: Type 1 diabetes
TOM: Topological overlap matrix
TSS: Transcription start site
UPR: Unfolded protein response
WGCNA: Weighted gene co-expression network analysis
ZFP57: ZFP57 zinc finger protein
