# Supplementary material for: Integrative genomic and transcriptomic analysis of genetic markers in Dupuytren’s disease
Source: BMC Med Genomics. 2019 Jul 11;12(Suppl 5):98. doi: 10.1186/s12920-019-0518-3 (PMC6624179; doi:10.1186/s12920-019-0518-3)
Supplement: Supplementary file 1 — Identification of soft-thresholding power for co-expression network construction. (A) An analysis of scale free topology for picking an appropriate soft-thresholding power. (B) An analysis of the mean connectivity for picking an appropriate soft-thresholding power. (PPTX 277 kb) [file 12920_2019_518_MOESM1_ESM.pptx]

## Slide 1
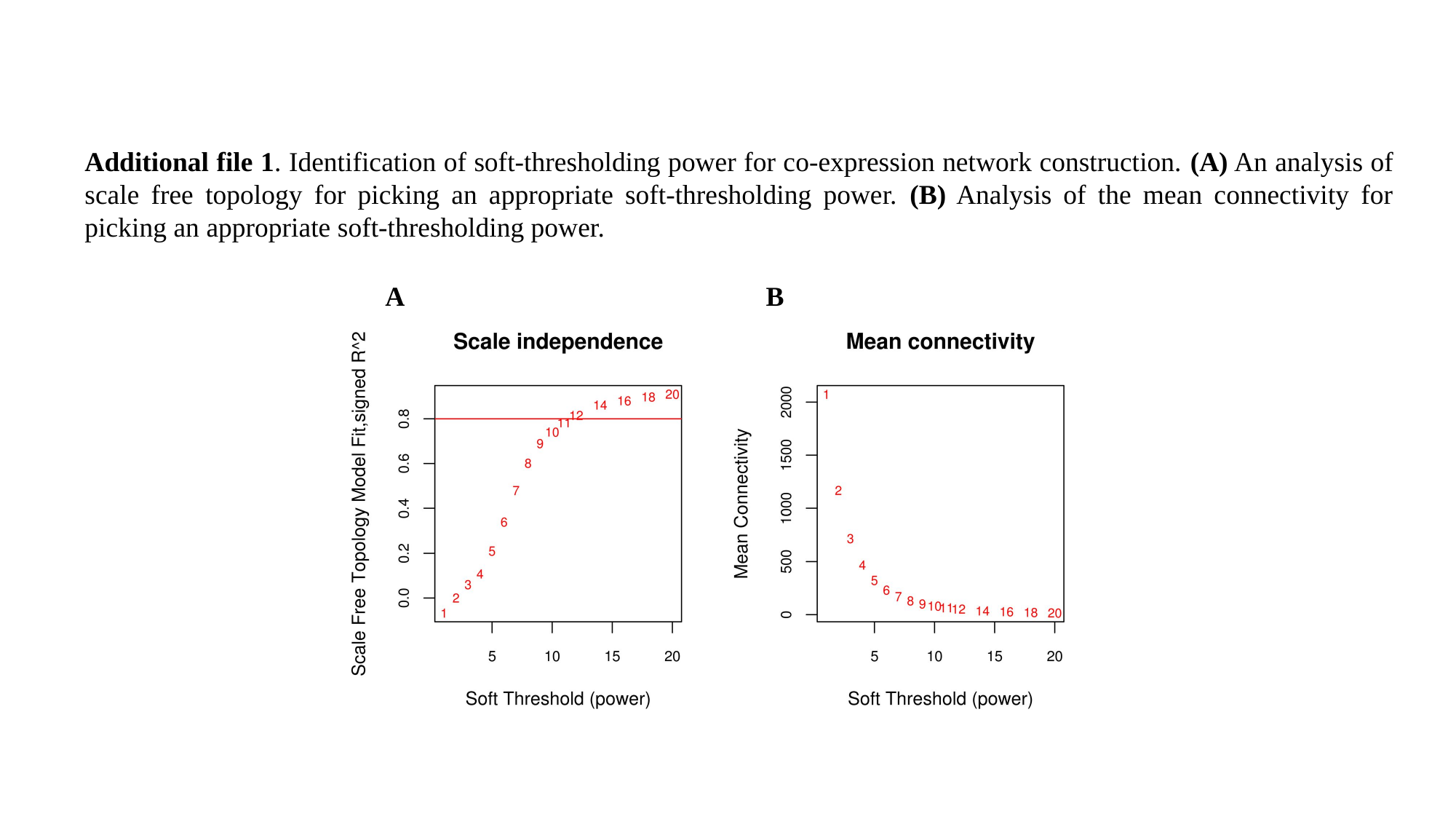

Additional file 1. Identification of soft-thresholding power for co-expression network construction. (A) An analysis of scale free topology for picking an appropriate soft-thresholding power. (B) Analysis of the mean connectivity for picking an appropriate soft-thresholding power.
A
B
