# Supplementary material for: Integrative genomic and transcriptomic analysis of genetic markers in Dupuytren’s disease
Source: BMC Med Genomics. 2019 Jul 11;12(Suppl 5):98. doi: 10.1186/s12920-019-0518-3 (PMC6624179; doi:10.1186/s12920-019-0518-3)
Supplement: Supplementary file 2 — Point range plots showing the results of the enrichment of DEGs about each module via Fisher’s exact test. (A) Odds ratios for the enrichment of DEGs compared with non-DEGs by one-color meta-analysis with 95% confidence intervals of odds. Because tan and midnight blue modules have no DEGs, the odds ratio is zero. (B) Odds ratios for the enrichment of DEGs compared with non-DEGs by two-color microarray analysis and 95% confidence intervals of the odds. (PPTX 242 kb) [file 12920_2019_518_MOESM2_ESM.pptx]

## Slide 1
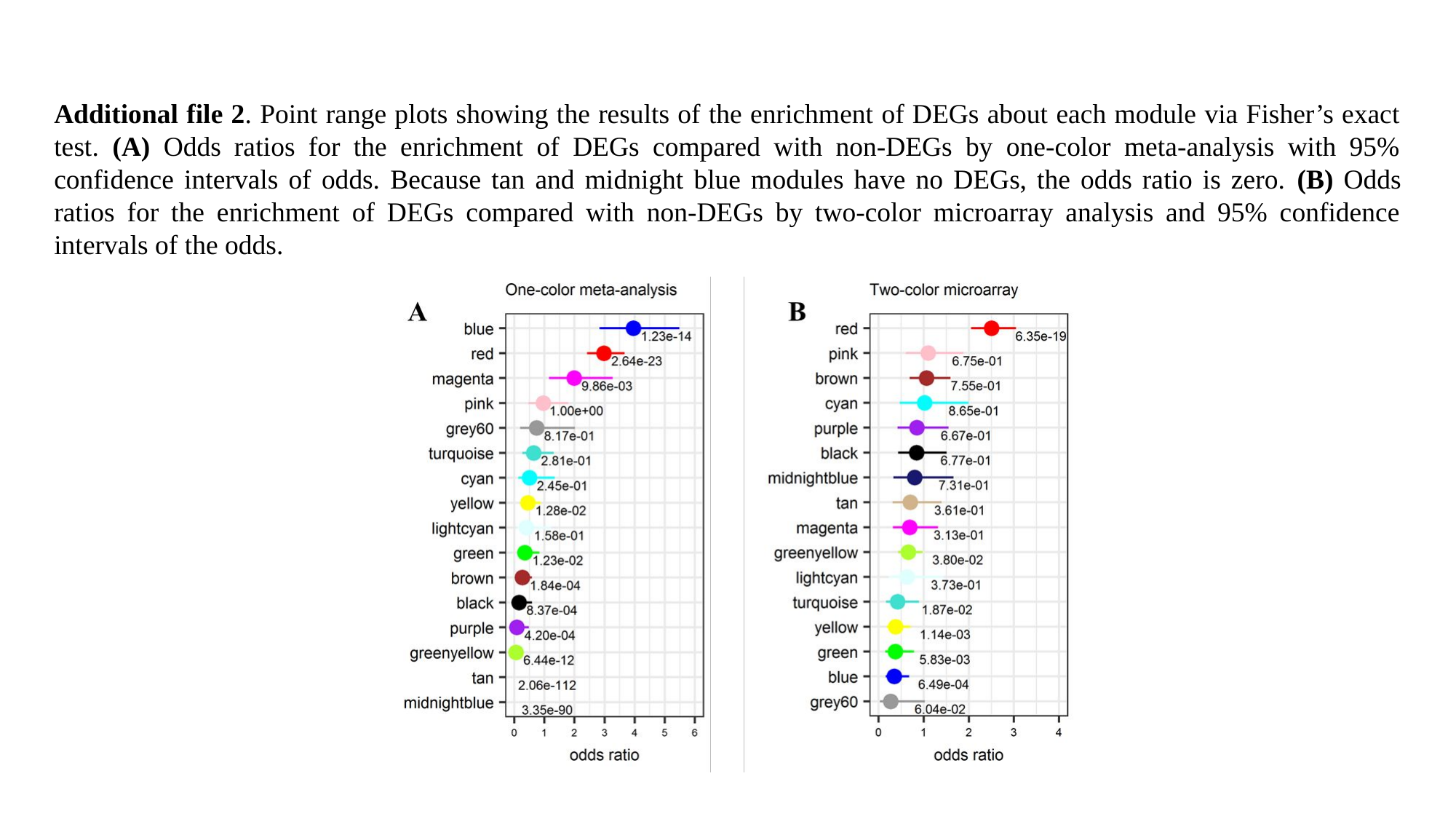

Additional file 2. Point range plots showing the results of the enrichment of DEGs about each module via Fisher’s exact test. (A) Odds ratios for the enrichment of DEGs compared with non-DEGs by one-color meta-analysis with 95% confidence intervals of odds. Because tan and midnight blue modules have no DEGs, the odds ratio is zero. (B) Odds ratios for the enrichment of DEGs compared with non-DEGs by two-color microarray analysis and 95% confidence intervals of the odds.
