# Supplementary material for: Integrative genomic and transcriptomic analysis of genetic markers in Dupuytren’s disease
Source: BMC Med Genomics. 2019 Jul 11;12(Suppl 5):98. doi: 10.1186/s12920-019-0518-3 (PMC6624179; doi:10.1186/s12920-019-0518-3)
Supplement: Supplementary file 5 — Violin plots showing the results of significant variant-gene associations related to rs2269423 in GTEx version 7. (PPTX 155 kb) [file 12920_2019_518_MOESM5_ESM.pptx]

## Slide 1
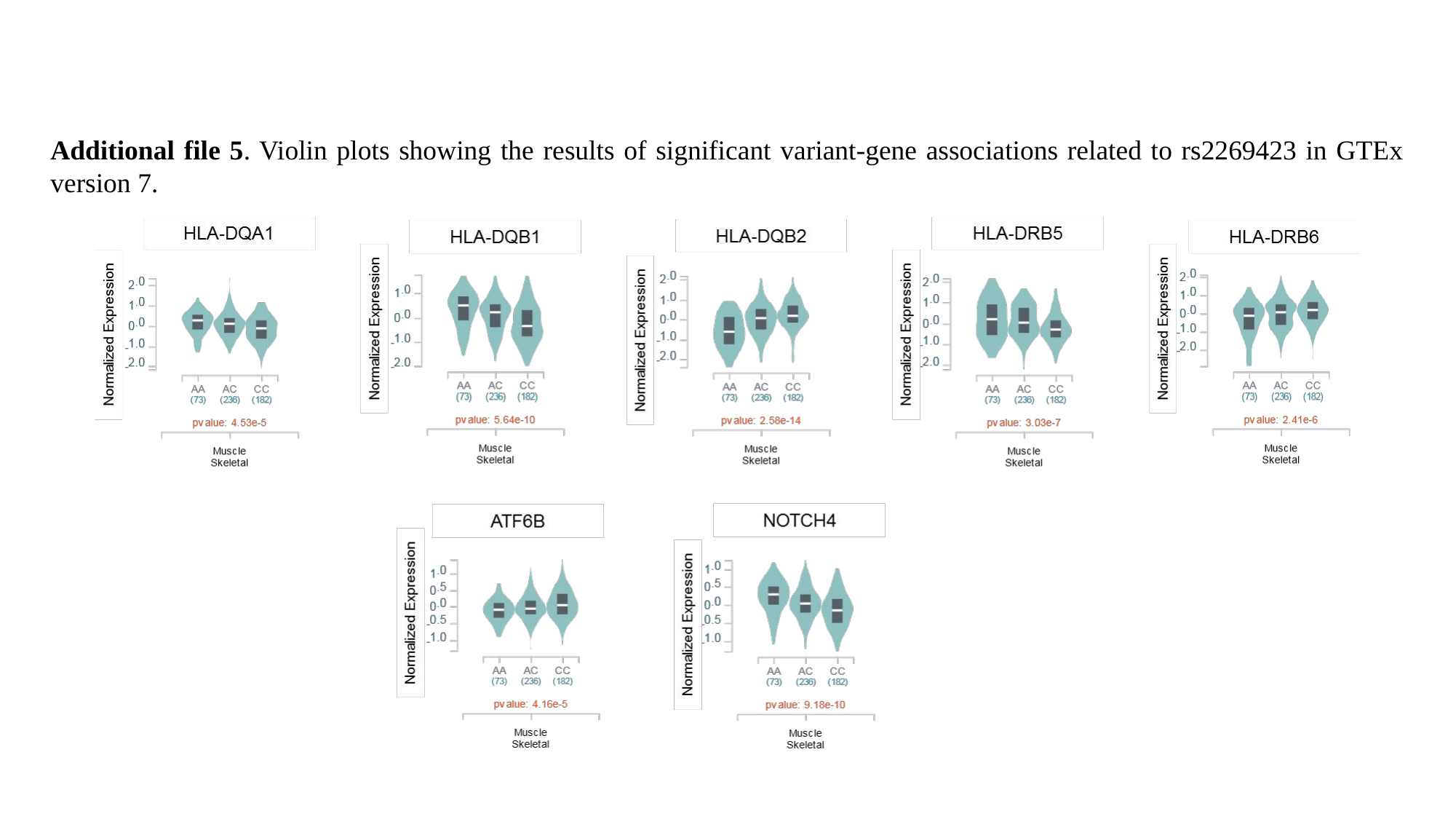

Additional file 5. Violin plots showing the results of significant variant-gene associations related to rs2269423 in GTEx version 7.
